# Scoliosis: density-equalizing mapping and scientometric analysis

**DOI:** 10.1186/1748-7161-4-15

**Published:** 2009-07-28

**Authors:** Karin Vitzthum, Stefanie Mache, David Quarcoo, Cristian Scutaru, David A Groneberg, Norman Schöffel

**Affiliations:** 1 Institute of Occupational Medicine, Charité-Universitätsmedizin Berlin, Free University Berlin and Humboldt-University Berlin, Thielallee 69-73, D-14195 Berlin, Germany; 2 Department of Respiratory Medicine, Hanover Medical School, Carl-Neuberg-Straß 1, 30625 Hanover, Germany

## Abstract

**Background:**

Publications related to scoliosis have increased enormously. A differentiation between publications of major and minor importance has become difficult even for experts. Scientometric data on developments and tendencies in scoliosis research has not been available to date. The aim of the current study was to evaluate the scientific efforts of scoliosis research both quantitatively and qualitatively.

**Methods:**

Large-scale data analysis, density-equalizing algorithms and scientometric methods were used to evaluate both the quantity and quality of research achievements of scientists studying scoliosis. Density-equalizing algorithms were applied to data retrieved from ISI-Web.

**Results:**

From 1904 to 2007, 8,186 items pertaining to scoliosis were published and included in the database. The studies were published in 76 countries: the USA, the U.K. and Canada being the most productive centers. The Washington University (St. Louis, Missouri) was identified as the most prolific institution during that period, and orthopedics represented by far the most productive medical discipline. "BRADFORD, DS" is the most productive author (146 items), and "DANSEREAU, J" is the author with the highest scientific impact (h-index of 27).

**Conclusion:**

Our results suggest that currently established measures of research output (i.e. impact factor, h-index) should be evaluated critically because phenomena, such as self-citation and co-authorship, distort the results and limit the value of the conclusions that may be drawn from these measures. Qualitative statements are just tractable by the comparison of the parameters with respect to multiple linkages. In order to obtain more objective evaluation tools, new measurements need to be developed.

## Background

Scoliosis is a disorder of increasing scientific interest. Since the first publication from 1904 has been entered in the ISI-Web database [1], annual publication numbers have risen exorbitantly, especially since 1990. Due to the bulk of publications, the scientific importance of particular items is difficult to rate, even after the implementation of the h-index and impact factor. These factors and publication numbers are often used as criteria to appraise a journal's, author's or institution's rank, although they do not exclude distorting factors like self-citation and co-authorship.

Scientometrics is a relatively new method to evaluate research accomplishments and their distributions. By comparing quantitative and qualitative factors, it is possible to evaluate this analysis methodically.

At the moment, there is no scientometric analysis on scoliosis available. The objectives of this study are:

1. To evaluate the distribution of publication numbers during a specific period of time, in a country-specific manner, and in terms of international cooperation, citation rates, publishing journals, authors, subject areas and institutions.

2. To apply well-established qualitative measures of citation analysis, impact factor and citation rates, to previously collected quantitative data and to reinterpret scientific efforts.

3. To clarify the scientific value of the currently established qualitative measures (i.e. h-index and the impact factor) in terms of scoliosis research.

## Methods

Data were retrieved from the Web of Science database, which is catalogued by the Thomson Institute for Scientific Information (ISI). ISI-Web was selected because of it offers complete reference data for all publications dealing with scoliosis. All abstracts of items included in this study were written in English.

All data files were examined with respect to the following variables: the country of origin, the publication date, medical disciplines, the institutions, the publishing journals and authors. To analyze the number of publications regarding the author, the data were transferred to Excel spreadsheets and presented as diagrams (see below).

In order to approximate the overall number of published items, the topic "scoliosis" was entered in the search field and combined with the Boolean operators, "AND" and "OR" using the word "scoliosis". Moreover, due to further aspects of the analysis, research in the database was refined by using both the "analyze" and the "citation report" functions. The time span analyzed covered the period from 1904, the first scoliosis-related publication in the database, to 2007. Results from 2008 and 2009 were not included in the analysis due to incomplete data acquisition.

By using the "citation report" method, the published items were analyzed to determine each country's number of citations per year and average citations per item (average citation rate). Further analysis of the average citation rate was performed following the exclusion of all countries with fewer than 30 publications. The average citation rate is used as an indicator for research quality [2-4]. It indicates the average number of cited articles for all items in the set and was calculated by dividing the number of citations per year by the number of results found each year.

The h-index is used as a qualitative measure of a scientist's merit. A scientist has index *h *if *h *of his or her *N *papers have at least *h *citations each and other (*N *- *h*) papers have, at most, *h *citations each [5]. Therefore, if a scientist has published ten articles, but only five of them were cited at least five times, his h-index is 5; if another scientist has published one article, which has been cited ten times, his h-index remains 1. The h-index is used in this paper to compare the most productive authors (measured by the number of published items) and determine the scientific impact his or her research in the scientific community.

The calculation of the journal impact factor is based on a two-year period. It is defined as the average number of citations in a given year of a journal's articles published over the two preceding years [3]. The impact factor is used in this paper to determine which journals are the most productive in the field of scoliosis research.

Density-equalizing mapping was used according to a recently published method [6,7], in which territories were resized according to a particular variable, i.e. the number of published items or the average citations per item. In this study, two maps depict the area of country rescaled in proportion to its total number of published scoliosis-related items and average citation rate, respectively. The specific calculations are based on Gastner's and Newman's algorithms [8]. Data acquired from ISI-Web revealed a systematic error in mapping the publications according to their country of origin, which was resolved by the following re-categorization. Publications from England, Northern Ireland, Wales and Scotland were collectively categorized as the United Kingdom (U.K.). Similarly, "Germany" referred to all papers from „WEST GERMANY“, "FED REP GER", „GER DEM REP“ and „BUNDES REPUBLIK“. Items published in other former countries, such as „Yugoslavia”, „Czechoslovakia” and the „USSR”, were examined with respect to the current country, in which the publishing institution is now located. Publications from „Czechoslovakia” were assigned to either "Slovakia" or the "Czech Republic". Likewise, publications from former „Yugoslavia” were assigned to Montenegro, Bosnia and Herzegovina, Croatia, Slovenia, Serbia and former Yugoslav Republic of Macedonia (FYROM). Publications from the former "USSR" were assigned to Belarus, Ukraine, Uzbekistan, Kazakhstan, Georgia, Azerbaijan, Lithuania, Moldova, Latvia, Kyrgyzstan, Armenia, Turkmenistan, Estonia, Tajikistan or Russia.

To evaluate the international cooperation between countries, reference data for the total number of publications were collected as plain text files and analyzed.

## Results

**Figure 1:**
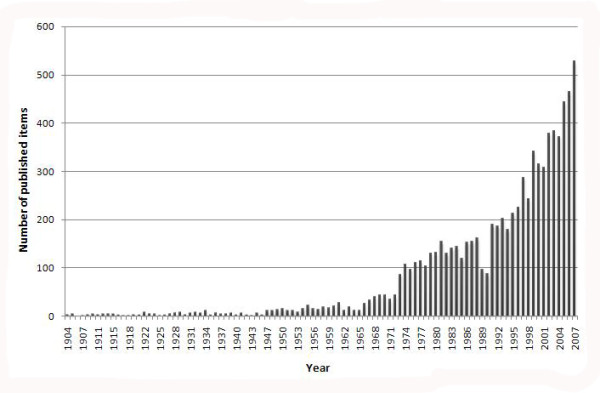
**Scoliosis-related publications in the Web of Sciences database from 1904 to 2007**.

The number of published items was used as a quantitative measure of research productivity. During the period from 1904 to 2007, a total of 8,186 items were published and indexed in ISI-Web. Since the late 1990's, frequency of publication has increased steeply (Fig. 1).

**Figure 2:**
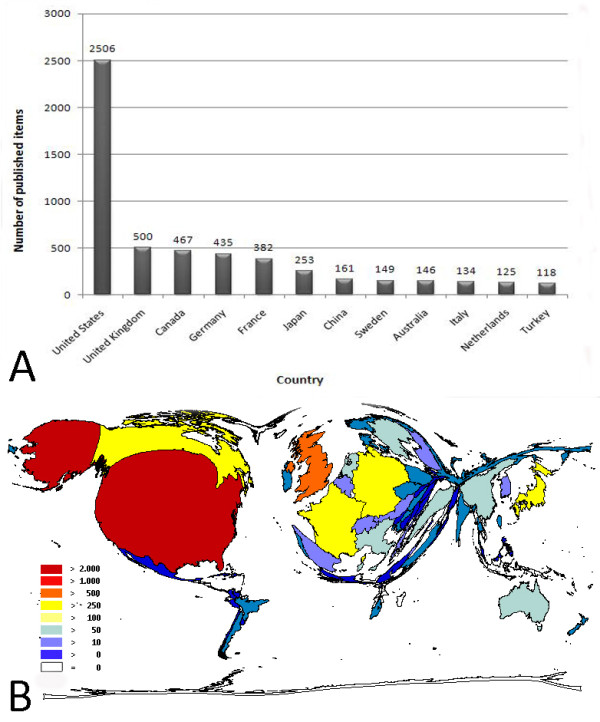
**A: Ranking of the total number of scoliosis-related publications per country**. Threshold of >100 published items. Study period from 1904 to 2007. 2B: Density-equalizing map illustrating the number of publications per country. The area of each country was scaled in proportion to its total number of scoliosis-related publications for period 1904 to 2007.

The 8,186 entries originated from 76 countries, of which the USA, the U.K. and Canada were the most productive countries, representing 42.4% of the publications (Fig. 2b). Twelve countries in the set had published more than 100 items (Fig. 2b). Density-equalizing mapping of this data set shows a tendency of a relatively small number of countries to publish a majority of the research (Fig. 2a).

**Figure 3:**
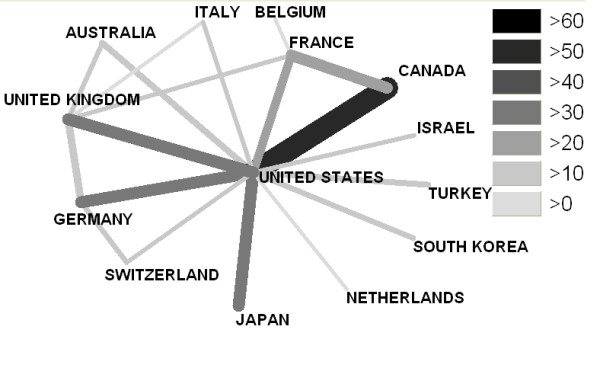
**Analysis of the international cooperation**. Threshold >10 cooperative partnerships.

In analyzing the reference data of all published items to determine the degree of international cooperation, the following partners were the most productive: U.S.A. and Canada (59), U.S.A. and Germany (33), U.S.A. and U.K. (32) and U.S.A. and Japan (32) (Fig. 3).

**Figure 4:**
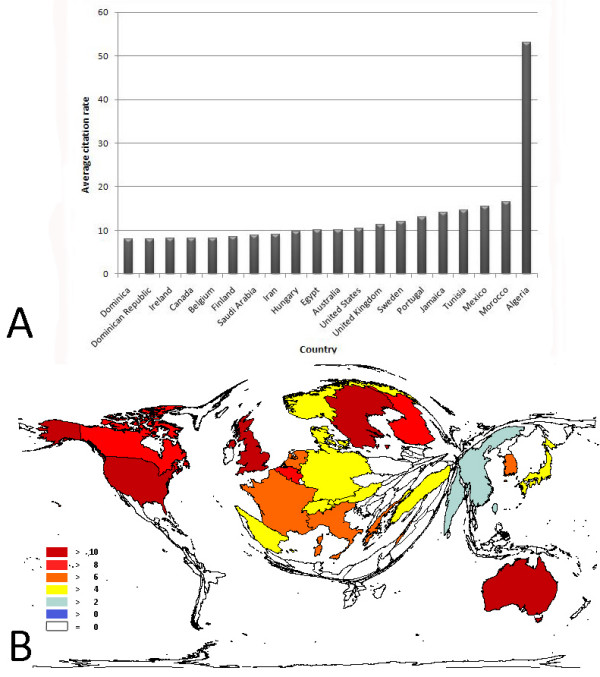
**A: Average citations per item rate in each of the 20 countries with the highest citation rates**. Study period from 1904 to 2007. 4B: Density-equalizing map showing the average citations per item in each country. Threshold excludes countries with <30 published items. The area of each country was scaled in proportion to its average citation rate. Study period from 1904 to 2007.

The highest citation rates among the countries in this data set were seen for Algeria (53.00), Morocco (16.57) and Mexico (15.40) (Fig. 4a). After excluding countries with 30 or fewer publications from the analysis, Sweden showed the highest citation rate (12.07), followed by the U.K. (11.29) and the U.S.A. (10.36) (Fig. 4b).

**Figure 5:**
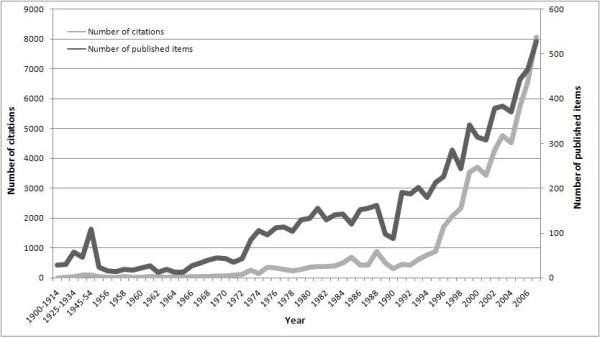
**Citations per year compared to the published items in each year**. Study period from 1904 to 2007.

To assess the changing degree of interest in scoliosis over the past century, data on the annual number of citations were analyzed for items published from 1904 to 2007. The data illustrated a trend of increasing citations since the beginning of the 1990's, which coincides with a general increase in articles published on scoliosis (Fig. 5).

**Figure 6:**
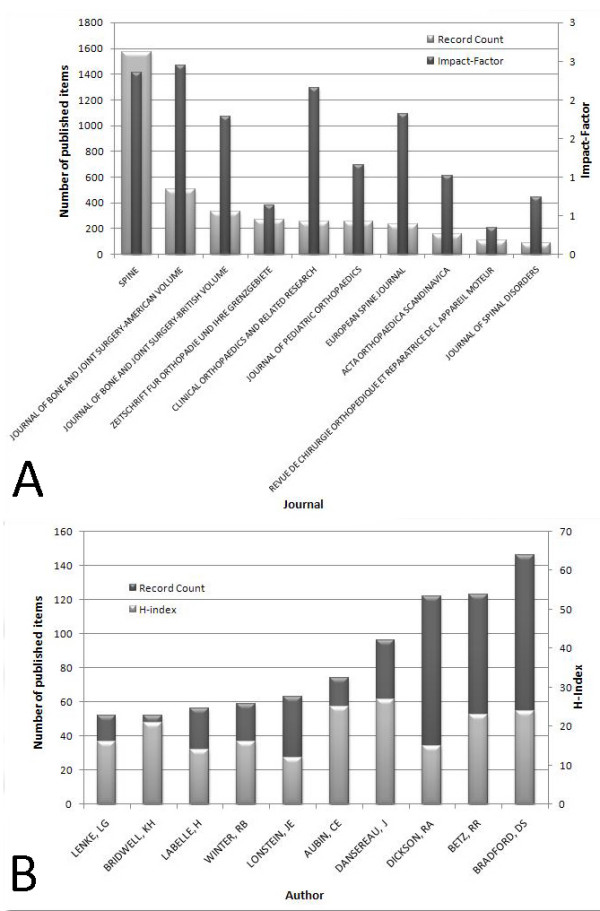
**A: Top ten ranking of journals by the number of publications and impact factor for items during the period from 1904 to 2007**. 6B: Ten most productive authors and their h-index in the period from 1904 to 2007.

The ten most productive journals for scoliosis research are listed in Fig. 6a. "SPINE" was the most productive with 1,573 published items, followed by the "JOURNAL OF BONE AND JOINT SURGERY-AMERICAN VOLUME" with 504 items, and the "JOURNAL OF BONE AND JOINT SURGERY-BRITISH VOLUME" with 337 items. The "JOURNAL OF BONE AND JOINT SURGERY-AMERICAN VOLUME" had the highest impact factor of 2.444, followed by "SPINE" with 2.351 and "CLINICAL ORTHOPAEDICS AND RELATED RESEARCH" with 2.161. Due to "SPINE's" high rankings for both publication number and impact factor, it should be considered the most prolific journal.

The most productive authors are presented in Fig. 6b. "BRADFORD, DS" is the most productive author with 146 items, followed by "BETZ, RR" with 123 items and "DICKSON, RA" with 122 items. "DANSEREAU, J" had the highest h-index of all authors (27), followed by "AUBIN, CE" (25) and "BRADFORD, DS" (24).

To evaluate scientific merit of the most important authors further, the number of published items (measure of quantity) and the h-index (measure of quality) were compared. Thus, "BRADFORD, DS" should be considered the most prolific author of scoliosis-related publications.

**Figure 7:**
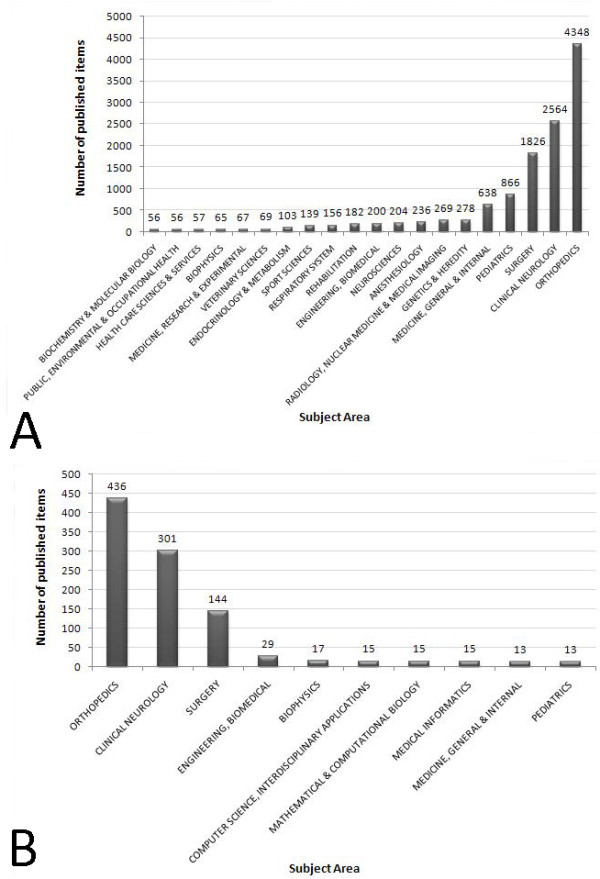
**A: Top twenty list of the most productive medical disciplines for scoliosis research**. Study period from 1904 to 2007. 7B: Most frequent medical disciplines of the ten most productive authors. Study period from 1904 to 2007.

The publications were sorted according to medical discipline. The most articles were published in the field of "ORTHOPEDICS" (4,348), followed by "CLINICAL NEUROLOGY" (2,564) and "SURGERY" (1,826) (Fig. 7a). Whereas the medical disciplines of the ten most productive authors were identical by rank, the next three entries showed a different distribution (Fig. 7b).

**Figure 8:**
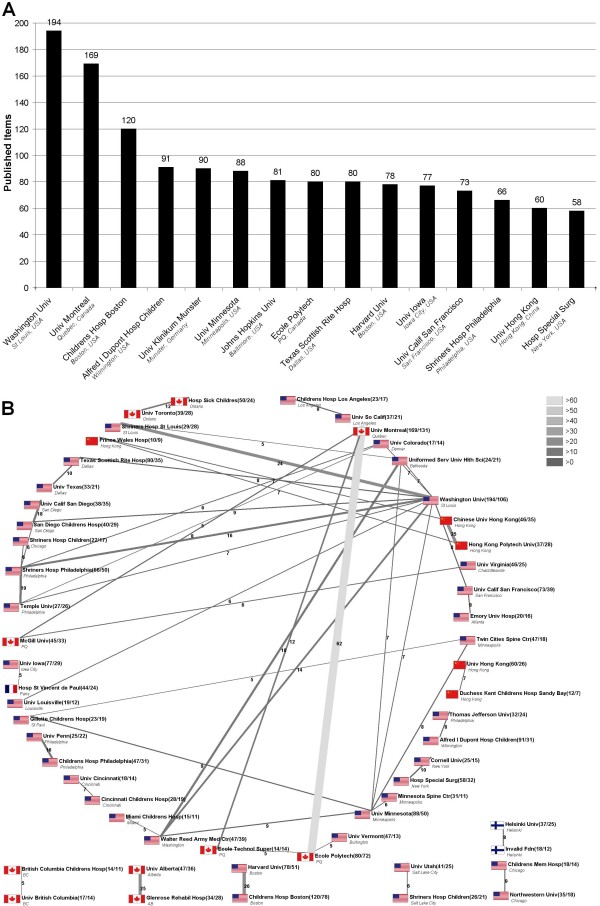
**A: Top fifteen ranking of the most productive institutions during the period from 1904 to 2007**. 8B: Analysis of institutional cooperation. Threshold excludes institutions with <10 published items.

The most productive institutions were the "WASHINGTON UNIV (St. Louis, Missouri)", "UNIV MONTREAL" and the "CHILDRENS HOSP BOSTON". Of the ten most productive institutions, four were not located in the U.S.A. (Fig. 8a) and the cooperation network visualizes the dominance of the connection between "ECOLE POLYTECHNIQUE" and the "UNIVERSITY OF MONTREAL" (Fig. 8b).

## Discussion

The present study was conducted to evaluate the scoliosis research output quantitatively and qualitatively using large-scale data analysis, scientometric approaches and density-equalizing procedures. An apparent increase in interest in scoliosis research has been seen as reflected by the constantly rising number of scoliosis-related publications since the beginning of the 1990's. However, this may largely be due to significant technical developments, such as availability of the internet-based scientific databases (storing abstracts in addition to title and keywords), new software programs (e.g. endnote) and online article submission, which accelerate the publication process. Data analysis in a country-specific manner showed that the USA, the U.K. and Canada maintained a leading position in scoliosis research. The tendency for a relatively small number of countries to publish the majority of items was clearly illustrated by density-equalizing mapping procedures. The sheer bulk of North American/Canadian and European publications caused a few countries virtually to disappear in the density-equalizing map. These data illustrate the dominant role of European and North American research in this field. By contrast, when analyzing the cooperation between particular countries, some countries, such as Turkey and South Korea, had detectable results, although they are not as well-established in the field. These results suggest that further research in the field of scoliosis may no longer be influenced predominantly by a small number of countries.

The ISI-Web database was selected for this analysis due to the availability of complete references of published items. These data built the basis for this analysis. By contrast, although PubMed includes similar numbers of published items, the corresponding reference data is incomplete. Otherwise it would have been interesting to compare findings of these two databases.

It would be of further interest to relate our findings on "scoliosis" to other orthopedic conditions and search for conflicting scientometric results and scientific tendencies.

The number of published items was used as one measure of quantitative research productivity. To evaluate the scientific impact of published items from several different countries, the average citation rate was determined for each country. We observed disproportionately high average citation rates among countries with a relatively small number of items. For instance, Algeria had an average citations rate of 53,00 (106 citations on 2 publications). Thus, a threshold of at least 30 published items chosen to be representative for inclusion in further analysis of countries' citation rates. The restricted analysis showed Sweden, the U.K., the USA and Australia to have the highest rates of citation.

Comparing the number of published items and international cooperation, the average citation rate for Australia and Sweden appeared disproportionately high. Further analysis revealed the tendency toward high self-citation rates in these two countries, which was also found for the USA, the U.K. and Finland. Moreover, increasing numbers of self-citation lead to above average citation rates and to the distortion of further qualitative research assessment [3-5,9]. In this respect, the value of established qualitative parameters such as impact factor h-index is limited.

To evaluate the most important authors of scoliosis-related publications, the number of published items (measure of productivity) and the h-index (measure of quality) were calculated. The results should be regarded skeptically due to the increasing tendency among authors within the past 15 years to self-cite and to co-author [4,10]. Calculations of the h-index still include self-citations and co-authorship, although these have an advantageous effect on the assessment of one's research output. In this context, the role of editors and reviewers and their influence on publishing processes could be of further interest. Interpreting our results it is apparent that the h-index and the journal's impact factor are not objective, independent indicators of research quality but, to a certain degree, susceptible to distortion by over-citation.

There might be some further bias concerning cooperating institutions. Certain institutes are affiliated with certain hospitals and authors might work for one or the other at different stages of publishing process or even at both institutions at the same time. Further studies should clarify author's scientific career progressions and work histories.

Although there have been no recent relevant advances in the diagnosis or in treatment of scoliosis [11], we expect an ongoing steep increase in scoliosis-related publications in the near future. It seems that modern scientific policies (pressure to keep one's position, to further one's career and secure one's monetary means) oblige scientists more to publish items than they can produce in terms of real scientific progress [4,10,12]. In light of these tendencies, it becomes necessary for researchers and clinicians to filter important findings from less important ones and prioritize their ongoing education, lest they risk being drowned by thousands of publications each year.

## Conclusion

The current study is the first detailed scientometric analysis of the importance and impact of the scoliosis research in science. The data show a marked increase in research productivity since the 1990's. While the majority of data originate from the U.S.A., the U.K. and Canada, Sweden takes a lead in the ranking of citations per item. Considering the scientific citation analysis, it can be assumed that there is an increasing interest in the topic. Nevertheless, analysis revealed that qualitative assessment according to established scientometric parameters (i.e. impact factor and h-index) should be considered critically because of self-citation and of co-authorship, which distort the results and limit the value of these measures. New qualitative measures should be established for more objective evaluation of scientific publications.

## Competing interests

The authors declare that they have no competing interests.

## Authors' contributions

All authors have read and approved the final version and the manuscript has not been submitted or published anywhere else. KV, NS and CS designed the study. NS and KV performed the search routines and constructed the different data files. SM, DQ and DAG performed pilot data search routines and analysis.
